# Utilization of Stiff Culm Trait of Rice *smos1* Mutant for Increased Lodging Resistance

**DOI:** 10.1371/journal.pone.0096009

**Published:** 2014-07-02

**Authors:** Ko Hirano, Ayako Okuno, Tokunori Hobo, Reynante Ordonio, Yusuke Shinozaki, Kenji Asano, Hidemi Kitano, Makoto Matsuoka

**Affiliations:** Bioscience and Biotechnology Center, Nagoya University, Nagoya, Aichi, Japan; Institute of Genetics and Developmental Biology, Chinese Academy of Sciences, China

## Abstract

Although the introduction of semi-dwarf trait into rice has led to improved lodging resistance making it capable of supporting high grain yield, lodging still remains a concern when attempting to further increase the grain yield of rice. However, improving the lodging resistance in rice by depending on the semi-dwarf trait alone is possible only up to a certain limit, beyond which other traits may be needed for reinforcement. To search for alternative traits relating to high lodging resistance, we identified 9 rice mutant lines possessing improved culm strength. To evaluate whether such lines can be useful for breeding lodging resistant rice, *small organ size1 (smos1)* mutant having increased lodging resistance but low tiller number and low grain yield, was chosen as a representative for a breeding trial. *smos1* was crossed with ST-4 (from the Stock rice collection of Nagoya University Togo field #4), a cultivar with high tiller number and high grain yield, and from their progeny, LRC1 (lodging resistance candidate-1) was selected. Although the low tiller number trait of *smos1* was not fully reversed in LRC1, this was compensated by an increase in grain weight per panicle, thereby resulting in high grain yield per plant. This important attribute of LRC1 was further enhanced by the improved lodging resistance trait inherited from *smos1*. Such improved lodging resistance in LRC1 and *smos1* was revealed to be mainly due to increased culm diameter and culm thickness, which led to a high section modulus (SM) value, a parameter defining the physical strength of the culm. Since *smos1* possesses high breaking-type lodging resistance which is different from semi-dwarf plants with high bending-type lodging resistance, an alternative approach of using thick culm lines for the creation of rice with increased lodging resistance is hereby proposed.

## Introduction

In the 1960’s, introduction of semi-dwarf trait into rice varieties combined with high nitrogen fertilizer input doubled rice grain yield, a success referred to as the “Green Revolution” [1]–[3]. Use of high amount of fertilizer allowed yield increase, while reducing plant height through the introduction of semi-dwarf gene increased lodging resistance, making rice capable of supporting grain-laden panicles. To avoid the future food crisis that may arise from human population explosion and climate change, it is urgent to increase rice grain yield per land area. In this context, lodging still remains a concern.

Lodging of cereal crops can be classified into three types [4]. Culm bending-type lodging is exhibited by plants when they cannot withstand the bending pressure, and is often observed in the upper internodes of rice during strong winds and rain. Culm breaking is another type of lodging that usually occurs at lower internodes (below the third internode counted from the top) as a result of excessive bending pressure at the higher internodes, and is determined primarily by the morphology and quality of the culm [5], [6]. The third type is root lodging, which results from the inability of the roots to support the above-ground part [7]. Root lodging is infrequently observed in transplanted rice, as they possess well-established root system.

The widespread use of gibberellin (GA)-related semi-dwarf plants during the “Green Revolution” was due to their improved bending-type lodging resistance. Short-statured plants have lower “center of gravity” than normal plants, which improves their resistance against bending pressure [8]. However, attaining lodging resistance through the use of GA-related semi-dwarf trait has some limitations. First of all, further improving the bending resistance does not necessarily result in increased lodging resistance. This is because although semi-dwarf plants have increased bending resistance in the culm, their breaking resistance is significantly reduced [9]. In addition, GA dependent semi-dwarf plants have lower grain yield than their original cultivars [9].

From the reasons mentioned above, a rather different approach is required to further improve lodging resistance in rice. One possible way is to increase the physical strength of the culm to improve breaking-type lodging resistance. This should satisfy the requirement for supporting higher grain yield without depending on semi-dwarfism.

In this study, we searched for rice mutants with stiff culm and improved breaking resistance, and 9 such lines were identified from more than 3000 rice mutant accessions. To investigate whether these traits could be utilized in the breeding program for improved lodging resistance, *small organ size1* (*smos1*) was chosen from the mutant lines and used for a breeding trial. Just recently, molecular characterization of *smos1* mutant revealed that it contains a mutation in a gene coding for APETALA2 (AP2)-type transcription factor which acts as an auxin-dependent regulator of cell expansion [10]. Thus, the mutation results in decreased ability of the cells to expand, hence, reducing the cell size in various organs of *smos1*. Surprisingly, the mutant also exhibits an increase in cell numbers in several organs which could account for *smos1*’s high lodging resistance. Although the molecular mechanism leading to increased cell numbers is unknown, expression of DNA replication-related genes is altered in *smos1* mutant. From such observation, Aya et. al. suggested one possibility that SMOS1 directly suppress cell proliferation through regulating these genes [10]. Unfortunately, the advantage of increased cell numbers of *smos1* is severely undermined by its poor number of tillers which translates into reduced grain yield per plant. Thus, as a crossing partner for *smos1*, we chose ST-4 (from the Stock rice collection of Nagoya University Togo field #4), a cultivar with high yield and large number of tillers. Through such a crossing combination, we succeeded to identify one progeny line possessing increased lodging resistance and grain yield. We hereby present that the stiff culm trait is yet another available option for breeding.

## Materials and Methods

### Growth conditions

Experiments were conducted at the Nagoya University Togo Field. Each rice line was planted in a 1×1 m plot with a planting density of 18 plants per square meter (15 cm horizontal and 30 cm vertical spacing between plants).

### Plant materials

*smos1* is a mutant derived from T65. ST-4 originated from inbred *japonica-indica* multiple crossing lines, but the parents were undeterminable. Crossing *smos1* with ST-4 was conducted using the latter as pollen donor. Among the self-fertilized F5 population of *smos1*×ST4 lines, a line tentatively named LRC1 (lodging resistance candidate-1), was selected for investigation. In the F5 lines, we did not observe any obvious differences within individual lines, suggesting that most of their genome regions were already fixed.

### Analyses of morphological and agronomical traits

For the measurement of each trait, plant samples were taken 40 days after heading, air-dried for 2 weeks, and analyzed.

### Analyses of lodging resistance

Lodging resistance parameters were measured from rice plants 40 days after heading. Culm bending resistance was investigated by measuring the cLr value (lodging resistance factor), following the method of Grafius and Brown [11]. Bending moment at breaking was measured at a distance of 4 cm between two supporting points as described previously [12]. Physical parameters were calculated using the formula: *M* = section modulus×bending stress [13]. *M* is the bending moment of the basal internode at breaking (g · cm). Section modulus (SM) = ∏/32×(a_1_^3^b_1_−a_2_^3^b_2_)/a_1_, where, a_1_ is the outer diameter of the minor axis in an oval cross-section, b_1_ is the outer diameter of the major axis in an oval cross-section, a_2_ is the inner diameter of the minor axis in an oval cross-section and b_2_ is the inner diameter of the major axis in an oval cross-section.

### Observation of the cross-section of the internode

The uppermost down to the fourth internode of rice plants at the matured seed stage were sampled. The center of each internode was hand-sectioned and stained with toluidine blue.

### Confirmation of *smos1* mutation by PCR

Genomic DNA of the F5 generation plants crossed with *smos1* and ST-4 were PCR amplified using the primers designed to amplify the *SMOS1* gene. Mutation was confirmed by sequencing the PCR amplified DNA.

### Statistical analysis

Data on morphological and agronomical traits, and lodging resistance were examined using three or more individual plants for replication. Statistical analyses were carried out using R version 2.12.1 software. Significance test was conducted using the two-tailed Student’s *t*-test or the Tukey’s significant difference test.

## Results

### Screening for rice mutants with improved lodging resistance parameters

**Figure 1:**
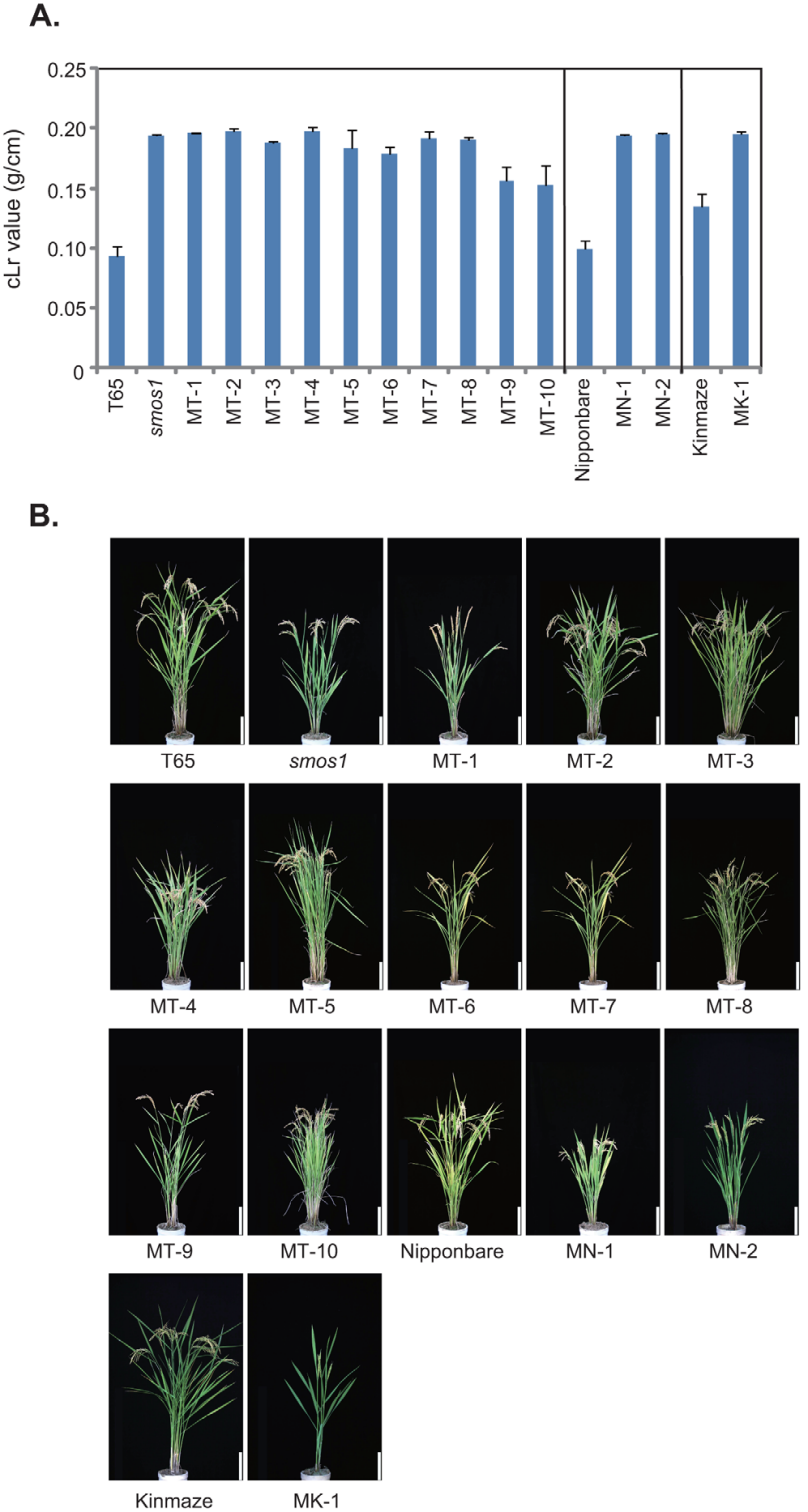
Rice lines with improved lodging resistance. (A) Bending-type lodging resistance of selected lines evaluated in terms of cLr value. Data are means ± SD (n> = 3). (B) Gross morphology of the selected lines. First up to the second panel of the third row show T65 (original cultivar) and T65 mutant lines. MN-1 and MN-2 at the third row are Nipponbare mutants and MK-1 at the fourth row is a Kinmaze mutant, respectively. Bar = 20 cm.

**Figure 2:**
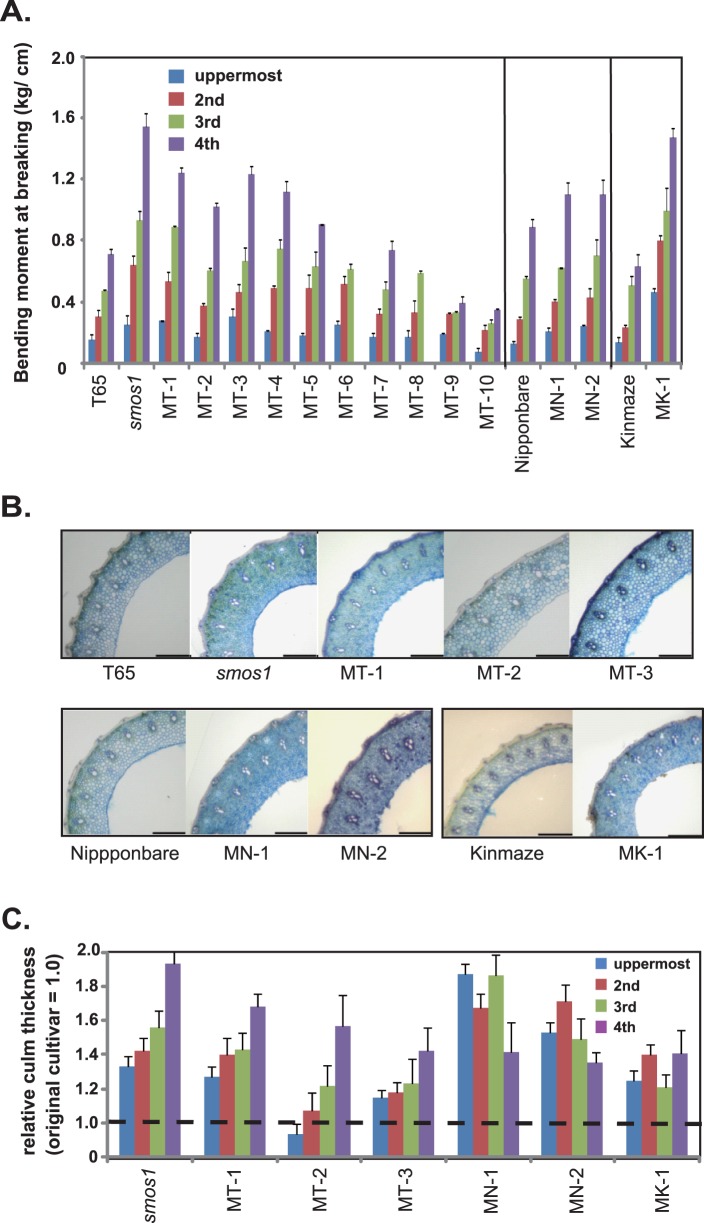
Breaking-type lodging resistance and culm morphologies of selected lines. (A) Breaking-type lodging resistance evaluated in terms of bending moment at breaking. Data are means ± SD (n> = 3). The fourth internodes of MT-6 and MT-8 could not be evaluated due to short internode lengths. (B) Magnified view of the third internode cross-section of 7 lines showing high bending-moment-at-breaking value in comparison with original cultivars (T65, Nipponbare, and Kinmaze). Bar = 500 µm. (C) Relative culm thickness of each line. Thickness of the original cultivars is set as 1. Data are means ± SD (n> = 3). The uppermost to fourth internodes were measured.

From among the >3000 rice mutant accessions of the Nagoya University Togo Field that we initially screened, we selected 14 lines showing improved cLr values (a measure for bending-type lodging resistance) for two seasons as compared to their respective original lines (T65, Nipponbare, and Kinmaze) (Figure 1A). Their gross morphologies are shown in Figure 1B. Next, we evaluated the bending moment at breaking (BMB, a measure for breaking-type lodging resistance) of the mutants (Figure 2A) using a high throughput handmade load-testing apparatus in the paddy field. Through these steps, we were able to classify the lines into two groups: 9 lines showed both increased cLr and BMB values, and 5 lines that only showed increased cLr values (MT-6 to -10).

Among the 9 mutants showing increased BMB, we further analyzed 7 lines, namely, *smos1,* MT-1, MT-2, MT-3, MN-1, MN-2, and MK-1 in terms of their culm morphology. The magnified view of the fourth internode of each culm is shown in Figure 2B. Almost all lines showed increased culm thickness in all the internodes tested as compared to original cultivars (Figure 2C).

Since our aim in this study is to find out whether there are other available options for breeding rice with improved lodging resistance other than the traditional use of semi-dwarf trait (which improves cLr), we decided to do a breeding trial using a rice line with improved BMB, while maintaining a good cLr value. For this purpose, we selected *smos1* from among the candidate mutant lines since it showed the highest BMB (Figure 2A). Previously, we identified the causal gene for *smos1* and found that it codes for an APETALA2 (AP2)-type transcription factor which controls organ size downstream of auxin signaling pathway [10]. Mutation in *smos1* results in reduced cell elongation but is accompanied by an increased cell number in several organs.

**Figure 3:**
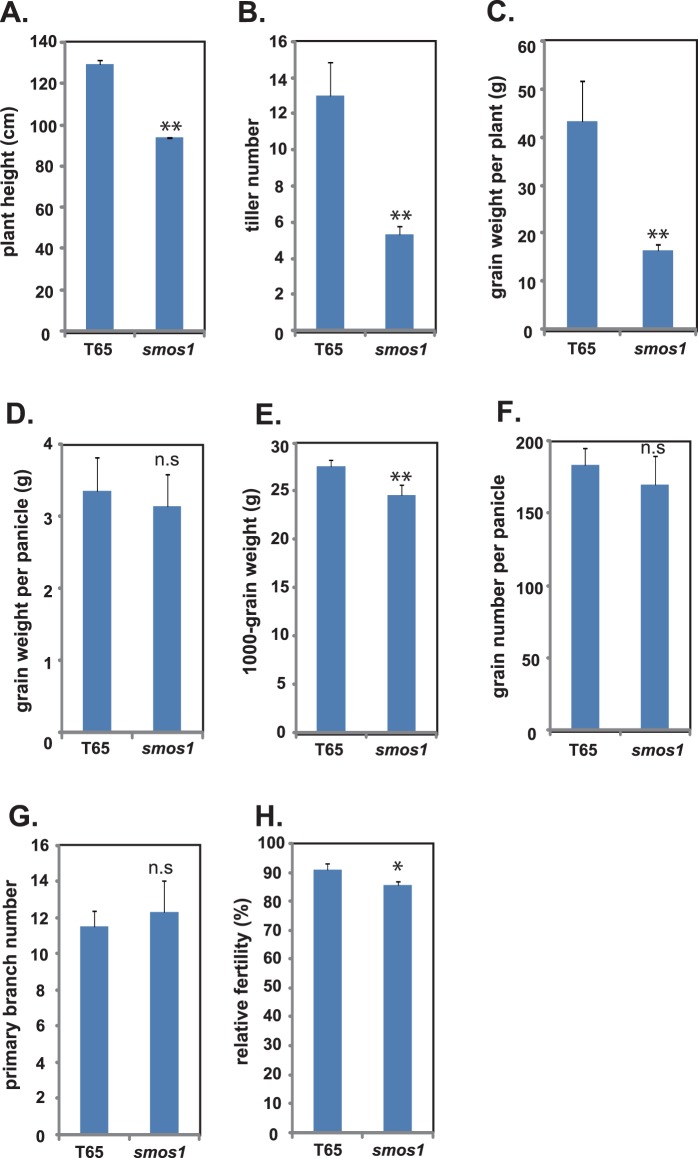
Plant height and agronomical traits of T65 and *smos1*. (A to H) Each trait of *smos1* was compared to that of its original cultivar T65. Data are means ± SD (n> = 3). **, ^n.s^, significantly different at P<0.01, and not significant, respectively (two-tailed Student’s *t*-test).

We initially evaluated the global agronomical attributes of *smos1* such as plant height, tiller number, grain weight per plant, grain weight per panicle, 1000-grain weight, grain number per panicle, primary branch number, and relative fertility in comparison to those of the original cultivar T65 (Figure 3). As a result, the plant height (Figure 3A), tiller number (Figure 3B), and total grain weight per plant (Figure 3C) of *smos1* were reduced to 72.0%, 40.8% and 38.1% relative to that of T65, respectively. As for the remaining grain-related traits, those of *smos1* did not vary significantly from that of T65, except for a slight 1000-grain weight and relative fertility reduction in *smos1* (Figure 3E and 3H; 11% and 5.6% reduction, respectively). Overall, this shows that the low tiller number of *smos1* is the main reason for the reduced grain weight per plant of this otherwise high yielding and lodging resistant line.

### Morphological traits of *smos1* x ST-4

**Figure 4:**
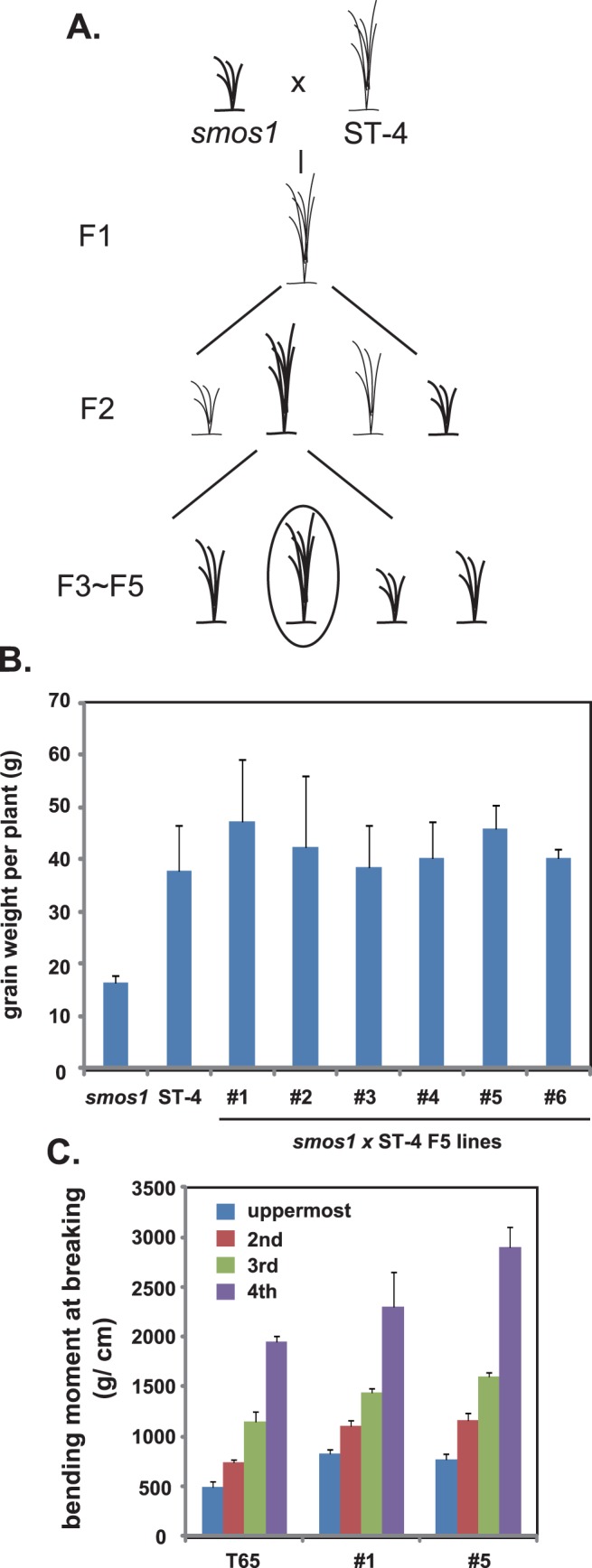
Selection of lines with high grain yield and improved lodging resistance from the F5 population of the *smos1* and ST-4 cross. (A) Procedure to select for high grain yield and improved lodging resistance. (B) Grain weight per plant of lines from the F5 population of the *smos1* and ST-4 cross. Data are means ± SD (n> = 3). (C) Bending-moment-at-breaking values of line #1 and line #5 (n> = 3).

For the breeding trial, we chose ST-4 as an ideal crossing partner to complement *smos1*’s unfavorable trait because of its high tiller number (14.3) and high grain yield (37.9 g of grain weight per plant). After crossing *smos1* with ST-4 (Figure 4A), the resulting F1 plant was self-fertilized to proceed to the next generation (F2). Such procedure was conducted until the F5 generation, each time selecting for lines with high grain yield and thick culm through visual and manual checking in the field. In the F5 generation, there were six independent lines (#1 to #6) that possess *smos1* mutant allele as confirmed by PCR, and showing similar or higher grain weight per plant relative to ST-4 (Figure 4B). Among them, we evaluated the BMB of the two highest yielding lines, #1 and #5, by using a precision load-testing machine (Tensilon RTM-25) for accurate measurement of BMB. Although both lines were superior to T65, #5 showed a larger BMB than #1 line (Figure 4C). Thus, we renamed #5 line as LRC1 (lodging resistance candidate-1) and studied it in more detail.

### Agronomical traits of LRC1

**Figure 5:**
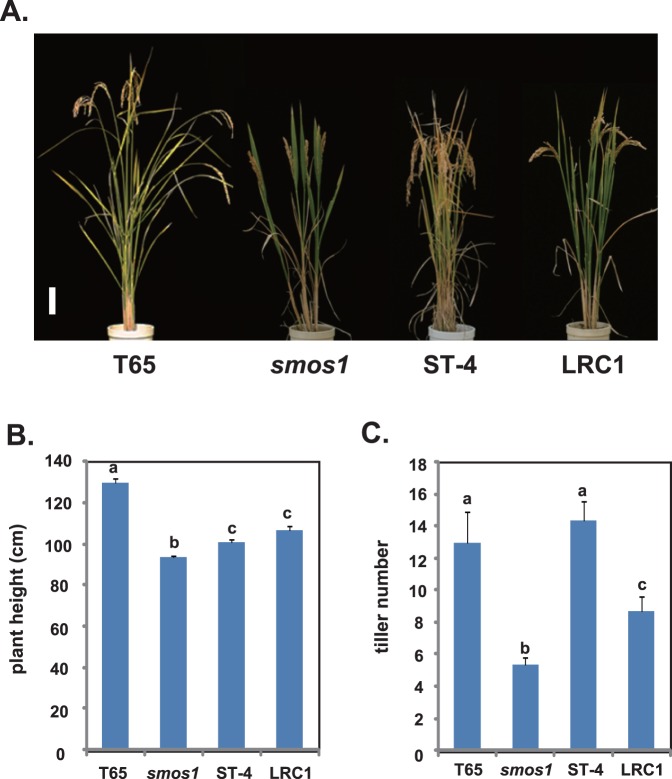
Morphology, plant height, and tiller numbers of T65, *smos1*, ST-4, and LRC1. (A) Gross morphology of plants at 30 days after heading. Bar = 10 cm. (B) Diagram for plant height. (C) Diagram for tiller numbers. Plant height and tiller numbers (n> = 3) were measured at 40 days after heading. Tukey’s test was conducted for panels (B) and (C).

Figure 5A shows the gross morphology of LRC1 and its parentals *smos1* and ST-4, together with T65. The plant height of LRC1 was slightly higher than that of *smos1* and similar to that of ST-4, whereas T65 was significantly higher than the three plants (Figure 5A and 5B). As mentioned, the tiller number of *smos1* was reduced compared to that of its original strain, T65 (Figure 5C). Compared to *smos1*, although LRC1 showed increased tillering with an average of 8.7, it was not fully restored to that of ST-4 (14.3) or T65 (13.0).

**Figure 6:**
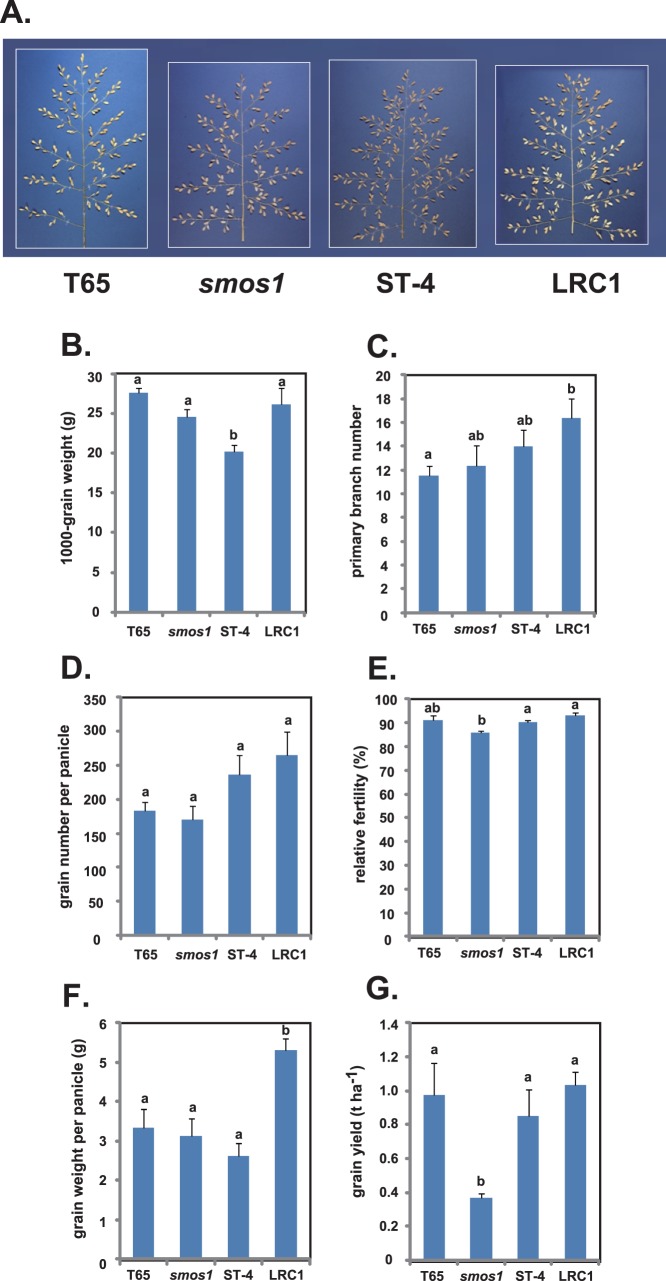
Panicle structure and yield-related traits of T65, *smos1*, ST-4, and LRC1. (A) The gross panicle morphology of each line at 40 days after heading. Diagrams for (B) 1000-grain weight, (C) primary branch number, (D) grain number per panicle, (E) relative fertility, (F) grain weight per panicle, and (G) grain yield per hectare are also shown. Data are means ± SD (n> = 3). Tukey’s test was conducted for panels (B) to (G).

Next, we evaluated the panicle structure of LRC1 (Figure 6). The 1000-grain weight of LRC1 (26.2 g) was similar to that of *smos1* (24.6 g) and T65 (27.6 g), whereas that of ST-4 was inferior (20.3 g; Figure 6B). On the other hand, the primary branch number (16.3) and grain number per panicle (264.7) of LRC1 were more similar to that of ST-4 (14.0 and 236.3, respectively) than to that of *smos1* (12.3 and 169.7, respectively; Figure 6C and 6D). Relative fertility was similar between the four plants, although *smos1* showed a slight decrease (Figure 6E). These data imply that LRC1 inherited the favorable 1000-grain weight trait from *smos1* and the high grain number and primary branch number traits from ST-4, which led to a marked increase in grain weight per panicle (5.3 g; Figure 6F) compared to the two parentals and T65 (2.6 to 3.3 g). Thus, despite the relatively low tiller number of LRC1, it achieved a grain weight per plant (shown as grain yield per hectare in Figure 6G) that was 20.1% and 5.7% higher than that obtained from ST-4 and T65, respectively, though these values are not statistically significant since the standard deviation for each plant was relatively high.

### Lodging resistance of LRC1

**Figure 7:**
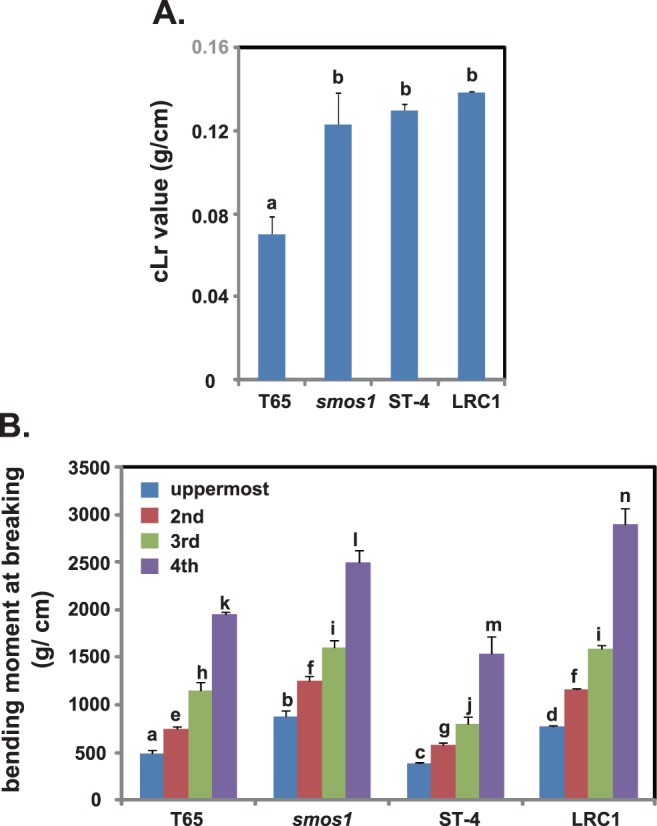
Comparison of lodging resistance between T65, *smos1*, ST-4, and LRC1. (A) Bending-type lodging resistance was analyzed in terms of cLr value. (B) Bending-moment-at-breaking lodging resistance. The uppermost to the fourth internode of each plant was used for analysis. Data are means ± SD (n> = 3). Tukey’s test was conducted for each panel.

Next, we evaluated the lodging resistance of LRC1 in terms of cLr and BMB values. The cLr value of LRC1 was similar to that of *smos1* and ST-4, but 1.9 times higher than that of T65 (Figure 7A). As for the BMB (Figure 7B), LRC1 had a value significantly higher than that of ST-4 and T65 in all the internodes analyzed, and *smos1* showed a similar trend. Thus, the improved lodging resistance of *smos1* was maintained in LRC1.

**Figure 8:**
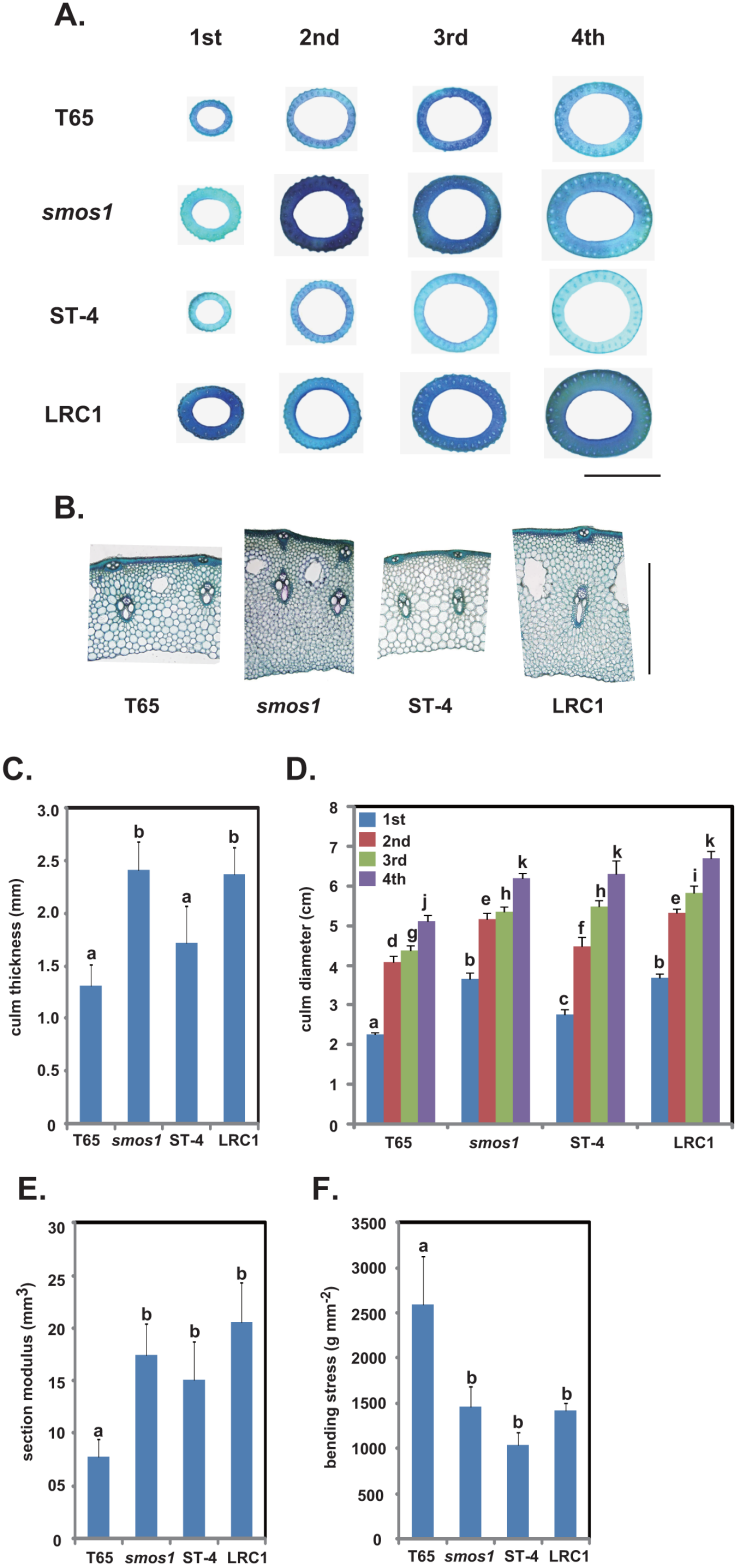
Culm morphology and culm strength of T65, *smos1*, ST-4, and LRC1. (A) Culm cross-sections from the uppermost (1st) to the fourth (4th) internodes of the main culms. Bar = 5 mm. (B) Cross-sections of the fourth internode showing the culm thickness of each plant. Bar = 1 mm. (C) Culm thickness of the fourth internodes of each plant (n = 3). (D) Culm diameter measured from the uppermost to the fourth internode of the main culms (n = 3). (E) Section modulus and (F) bending stress of each plant (n = 3). Tukey’s test was conducted for panels (C) to (F).

BMB can be further subdivided into two parameters. That is, the section modulus (SM), which is determined by the morphology of the culm (thickness and diameter), and the bending stress (BS) coefficient, which is influenced by the quality of the culm such as the amount of cellulose and lignin within the cell wall [12]. To investigate the mechanism behind the improved culm stiffness of *smos1* and LRC1, culm thickness, culm diameter, SM, and BS were measured at the fourth internodes of each line (Figure 8). Initially, we observed the transverse sections of the uppermost to the fourth internode of *smos1* and LRC1 and found that they are apparently different from those of T65 and ST-4 for having thicker culms (Figure 8A). When the fourth internode transverse sections were observed under the microscope, those from the culms of *smos1* and LRC1 possessed smaller but increased number of cells (Figure 8B), resulting in 1.8 and 1.4 (*smos1*) times, and 1.9 and 1.5 (LRC1) times culm thickness compared to T65 and ST-4, respectively (Figure 8C). When culm diameter, another parameter determining the SM was measured, *smos1* and LRC1 showed a significantly larger diameter compared to T65 in all the internodes analyzed (Figure 8D). *smos1* and LRC1 showed a similar or slightly higher diameter compared to ST-4.

As was expected from their culm morphology, SMs of the fourth internodes of *smos1* and LRC1 were 2.3 and 2.7 times higher than that of T65, respectively (Figure 8E) and although not significant, the SM of LRC1 was higher than that of *smos1*. This might be the reason why LRC1 showed a slightly higher BMB at the fourth internode relative to *smos1* (Figure 7B).

As for the BS of each plant (Figure 8F), BS of *smos1* (and LRC1) was much lower than that of T65 (Figure 8F). This suggests that there might be a trade-off between SM and BS, and that pronounced increase in culm thickness and diameter negatively affects culm quality that in turn decreases BS (yet to be proven).

Taken altogether, the increased breaking-type lodging resistance of *smos1* and LRC1 results from the higher SM conferred by their thick and wide culms, which are lacking in T65.

## Discussion

In the present study, we showed that some mutants with stiff culm have the potential to markedly increase breaking-type lodging resistance (Figure 2A). However, as far as we know, high BMB mutants have not been utilized in breeding programs up to now. This is probably due to the various unfavorable traits that are often associated with these lines, such as low tiller number, low grain yield, and some morphological defects. To assess the possibility of utilizing mutants with improved culm breaking resistance in breeding, we chose *smos1* as a representative mutant line possessing increased lodging resistance but with reduced tillers and low grain yield. As a crossing partner, we chose ST-4, a high tillering and high grain-yielding line that has inferior breaking-type lodging resistance, to investigate the possibility of producing rice with high lodging resistance and high grain yield.

### Selecting rice line with stiff culm and restored tiller number

The incomplete restoration of the tiller number of LRC1 to that similar to ST-4 suggests that it is difficult to separate traits related to culm thickness (or size) and tiller number, and that the two traits may be pleiotropic effects of the same mechanism(s). Such negative correlation between tiller number and culm size has also been reported. For instance, *fine culm 1* (*fc1*) mutant shows high tiller number which results from diminished strigolactone signaling, has thin culms [14]. Similarly, *high-tillering dwarf 3* mutant possesses small culm diameter although its mechanism is unknown [15]. These observations suggest that there is a trade-off between tiller number and culm size.

Even so, the significant improvement of *smos1* in terms of lodging resistance is an attractive trait that can be harnessed in rice breeding programs. One possible way to overcome the negative effect of low tiller number is to increase grain weight per panicle. In Figure 6F, the observation that LRC1 had a significantly higher grain weight per panicle (5.3 g) than any of its parents (ST-4, 2.6 g; *smos1*, 3.1 g) or T65 (3.3 g) shows that provided a suitable crossing partner is used with *smos1* and selection of best-performing progenies is done, good candidates with thick culm and a reasonable grain yield can be obtained.

### Using lines with unfavorable traits– a paradigm shift in rice breeding

Although numerous researches have been devoted to increasing rice grain production, a substantial increase in yield has been slow in coming after the “Green Revolution” [3]. This calls for new strategies to overcome the current rice production bottleneck and possibly bring forth a Second Green Revolution. As shown in this study, a mutant line (*smos1*) that have been ruled out as a possible breeding line in the past due to its unfavorable trait surprisingly gave rise to a lodging resistant and high yielding progeny (LRC1) after crossing with ST-4. This means that even lines with unfavorable traits from a certain viewpoint can serve as important bioresources and that a paradigm shift in the way we look at them is important.

## References

[1] Hargrove TR, Cabanilla VL (1979) The impact of semi-dwarf varieties on Asian rice-breeding programs. BioScience 29: 731–735.

[2] Dalrymple DG (1986) Development and spread of high-yielding rice varieties in developing countries. International Rice Research Institute.

[3] Khush GS (1999) Green revolution: preparing for the 21st century. Genome 42: 646–655. 10464789

[4] Kono M (1995) Physiological aspects of lodging. In: Matsuo T, Kumazawa K, Ishii R, Ishihara K, Hirata H, eds. Science of the rice plant, Volume 2, Physiology. Tokyo: Food and Agriculture Policy Research Center 971–982.

[5] Hoshikawa K, Wang SB (1990) Studies lodging in rice plants. I. A general observation on lodged rice culms. Jpn J Crop Sci 59: 809–814.

[6] Islam MS, Peng S, Visperas RM, Ereful N, Bhuiya MSU, et al (2007) Lodging-related morphological traits of hybrid rice in a tropical irrigated ecosystem. Field Crops Res 101: 240–248.

[7] Watanabe T (1997) Lodging resistance. In: Matsuo T, Futsuhara Y, Kikuchi F, Yamaguchi H (eds) Science of the rice plant, vol 3, Genetics, vol 3. Food and Agriculture Policy Research Center, Tokyo, 567–577.

[8] Kashiwagi T, Hirotsu N, Madoka Y, Ookawa T, Ishimaru K (2007) Improvement of resistance to bending-type lodging in rice. Japan Journal of Crop Science 76: 1–9.

[9] Okuno A, Hirano K, Asano K, Takase W, Masuda R, et al (2014) New approach to increasing rice lodging resistance and biomass yield through the use of high gibberellin producing varieties. Plos One 19 9: e86870. 10.1371/journal.pone.0086870 PMC3929325 24586255

[10] Aya K, Hobo T, Sato-Izawa K, Ueguchi-Tanaka M, Kitano H, et al.. (2014) A novel AP2-type transcription factor, SMALL ORGAN SIZE1, controls organ size downstream of an auxin signaling pathway. Plant Cell Physiol. 2014 PMID: 24486766. 10.1093/pcp/pcu023 24486766

[11] Grafius JE, Brown HM (1954) Lodging resistance in oats. Agronomy Journal 46: 414–418.

[12] Ookawa T, Ishihara K (1993) Varietal difference of the cell wall components affecting the bending stress of the culm in relation to the lodging resistance in paddy rice. Japan Journal of Crop Science 62: 378–384.

[13] Ookawa T, YasudaK, Kato H, Sakai M, Seto M, et al (2010) Biomass Production and Lodging Resistance in ‘Leaf Star’, a New Long-Culm Rice Forage Cultivar. Plant Prod Sci 13: 56–64.

[14] Takeda T, Suwa Y, Suzuki M, Kitano H, Ueguchi-Tanaka M, et al (2003) The OsTB1 gene negatively regulates lateral branching in rice. Plant J. 33: 513–520. 10.1046/j.1365-313x.2003.01648.x 12581309

[15] Zhang B, Tian F, Tan L, Xie D, Sun C (2011) Characterization of a novel high-tillering dwarf 3 mutant in rice. J Genet Genomics 38: 411–418. 21930100 10.1016/j.jgg.2011.08.002

